# An Editorial for the Special Issue “Actinoids in Biologic Systems and Catalysis”

**DOI:** 10.3390/molecules29122892

**Published:** 2024-06-18

**Authors:** Dongqi Wang

**Affiliations:** School of Chemistry, Dalian University of Technology, Dalian 116024, China; wangdq@dlut.edu.cn

The recent few decades witnessed a quick growth in our knowledge in actinoid chemistry, particularly in actinoids’ behaviors in catalysis and biologic systems. This knowledge is important to the sustainable civil application of nuclear fission energy and contributes to an objective evaluation of potential influence to the environment and health. Studies in these two issues have touched upon the fundamental nature of coordination chemistry of actinoids, and our knowledge has shaped new views around them, offering extensive influence on their civil application.

On this occasion, we are delighted to introduce a Special Issue to acknowledge the advancements. In this Special Issue, we are honored to invite nine contributions that cover the catalytic activity and migration behavior of f-block elements in the environment and biosystems.

In this Special Issue, the following contributions were collected: Wang et al. Contribution 1 studied the influence of light on the migration behavior of uranyl and found that it can promote the immobilization of uranyl(VI) using ferrihydrite. Li et al. Contribution 2 carried out a relativistic DFT study to reveal the catalytic reactivity of uranium-doped zinc, copper, and nickel oxides in the conversion of furfural to furfuryl alcohol, finding that these metal oxides can enhance catalytic reactions. Gui et al. Contribution 3 conducted a study to reveal the effects of CeO_2_ nanoparticles on the nutritional quality of two crop plants, corn (*Zea mays* L.) and soybean (*Glycine max* L.). Wang et al. Contribution 4 reported the folding dynamics of 3,4,3-LI(1,2-HOPO), a potential decorporation agent to remove in vivo actinoids, in its free and bound state with U^4+^ by means of MD simulations. Chen et al. Contribution 5 investigated the role of the lateral dimensional property of graphene oxide on its interactions with renal cells. Tian et al. Contribution 6 studied the biochemistry of nano-WSe_2_ and identified that it could be absorbed and transformed by rice plants.

This Special Issue also contains three review articles. Bao et al. Contribution 7 reviewed the sorption and selective extraction of actinoids from aqueous solutions. Zhong et al. Contribution 8 studied the analytical methods for the determination of ^90^Sr and ^239,240^Pu in environmental samples. Wang et al. Contribution 9 investigated recent advances in the study of extracting trivalent lanthanides and actinoids using phosphinic and thiophosphinic ligands in condensed phases.

We acknowledge the authors’ contributions and dedicate this Special Issue to Prof. Zhifang Chai to honor his contributions to the research and discipline development of radiochemistry.

